# A survey of *Mycoplasma agalactiae* in dairy sheep farms in Spain

**DOI:** 10.1186/1746-6148-8-171

**Published:** 2012-09-24

**Authors:** Jaime Ariza-Miguel, David Rodríguez-Lázaro, Marta Hernández

**Affiliations:** 1 Instituto Tecnológico Agrario de Castilla y León (ITACyL), Consejería de Agricultura y Ganadería. Junta de Castilla y León, Carretera de Burgos km. 119, C.P. 47071, Valladolid, Spain

**Keywords:** Mycoplasma agalactiae, Contagious agalactia, Real time PCR, Sheep, Dairy, Spain

## Abstract

**Background:**

Contagious Agalactia (CA) is one of the major animal health problems in small ruminants because of its economic significance. Currently, four *Mycoplasma* spp. have been associated with this syndrome: *M. agalactiae*, *M. mycoides* subsp. *capri*, *M. capricolum* subsp. *capricolum* and *M. putrefaciens*. Their presence has been evaluated in several studies conducted in CA-endemic countries. However, previous Spanish studies have been focused on caprine CA, and there is a knowledge gap regarding which *Mycoplasma* species are present in sheep flocks from Spain, which has the second highest number of sheep amongst the 27 European Union member states. Consequently, we investigated the presence and geographic distribution of the four CA-causing mycoplasmas in Spanish dairy sheep farms. This is the first time such an investigation has been performed.

**Results:**

Three hundred thirty nine out of 922 sheep flocks were positive for *M. agalactiae* by real time PCR (36.8%) and 85 by microbiological identification (9.2%). Interestingly, all 597 milk samples assessed for the presence of *M. mycoides* subsp. *capri*, *M. capricolum* subsp. *capricolum* and *M*. *putrefaciens* tested negative. To evaluate the intermittent excretion of the pathogen in milk, we sampled 391 additional farms from 2 to 5 times, resulting that in 26.3% of the cases a previously positive farm tested negative in a later sampling.

**Conclusions:**

*M*. *agalactiae* was the only *Mycoplasma* species detected in the study area showing a high frequency of presence and wide distribution. Therefore, the establishment of a permanent surveillance network is advantageous, as well as the implementation of control and prevention measures to hinder the dissemination of *M. agalactiae* and to prevent the entrance of other *Mycoplasma* species.

## Background

Contagious Agalactia (CA) is, along with bovine pleuropneumonia and contagious caprine pleuropneumonia, one of the three Mycoplasma-induced diseases affecting small ruminants which are notifiable to the World Organisation for Animal Health due to their economic significance [1]. CA is a syndrome clinically characterized by mastitis, arthritis, keratoconjunctivitis and occasionally abortion [2,3] and *Mycoplasma agalactiae* is considered its major etiological agent. *M*. *mycoides* subsp. *capri*, *M*. *capricolum* subsp. *capricolum* and *M*. *putrefaciens* cause a clinically similar syndrome, particularly in goats [4,5]. The syndrome causes major economic losses because of reduction or suppression of milk production, abortion, high morbidity and mortality rates, and costs associated with the diagnosis, treatment and prevention which are estimated to be above 20 million Euros per year in the European countries forming the Mediterranean Basin [3,4,6].

CA has its major impact in the Mediterranean countries, where the disease is considered to be endemic. However, it is also widely distributed in west Asian countries, central, north and east African Countries, the USA, and Brazil [1,2,7-9]. Interestingly, the significance of the different *Mycoplasma* species causing CA varies depending on the geographic area. In the United States, *M. mycoides* subsp. *capri* is the most prevalent caprine *Mycoplasma*, although *M. agalactiae* has been recently isolated [9]. In Northern Jordan, *M. agalactiae* and *M. mycoides* subsp. *capri* play the major role in both, sheep and goats [7,10]. In France, *M. agalactiae* has reemerged in sheep flocks located in the basin of the Western Pyrénées, causing 98 new outbreaks in 2008 [1]. In Spain, which has the second highest number of sheep amongst the 27 European Union member states, research has been focused on caprine CA, and there is a knowledge gap regarding which *Mycoplasma* species are present in sheep flocks from that country.

Consequently, in the present study we aimed to assess for the first time the presence and geographic distribution of the four *Mycoplasma* species causing CA, by analyzing raw milk samples from Spanish dairy sheep farms by classical microbiological methods, and PCR-based methods which have been demonstrated to be specific and sensitive [11-13]. The knowledge acquired will allow the implementation of appropriate control programs for those pathogens.

## Results

### Sensitivity of the PCR-based methods and capability to detect coinfected samples

Artificially contaminated milk samples (50 mL) inoculated with from 10^8^ cells to 10^2^ cells of each *Mycoplasma* gave positive results by the three PCR-based systems [11-13]. Detection failed in the milk samples seeded with 10^1^ cells (0.2 cells/mL). Capability to detect samples co-infected with various CA-causing mycoplasmas was demonstrated in samples artificially contaminated with the 4 *Mycoplasma* species analyzed in this study.

### Detection of CA-causing mycoplasmas in Spanish sheep farms

All 597 milk samples tested negative for *M. mycoides* subsp. *capri*, *M. capricolum* subsp. *capricolum* and *M*. *putrefaciens*. On the other hand, 339 out of 922 dairy sheep farms were positive for *M*. *agalactiae* by real time PCR (36.8%). Furthermore, the pathogen was observed by microscopy identification in samples collected from 85 flocks (9.2%). Thereby, 411 *M*. *agalactiae* isolates were obtained.

We assessed the level of intermittent excretion of *M. agalactiae* during the sampling period using data obtained from other additional 391 farms sampled repeatedly from 2 to 5 times. In 26.3% of the cases a previously positive farm resulted negative in a later sampling and conversely, 38 farms (9.7%) tested negative in the first sampling but resulted positive in any of the subsequent samplings. Overall, 250 of those sheep farms (63.9%) were positive for *M*. *agalactiae*.

### Geographic distribution of flocks infected with *Mycoplasma agalactiae*

**Figure 1:**
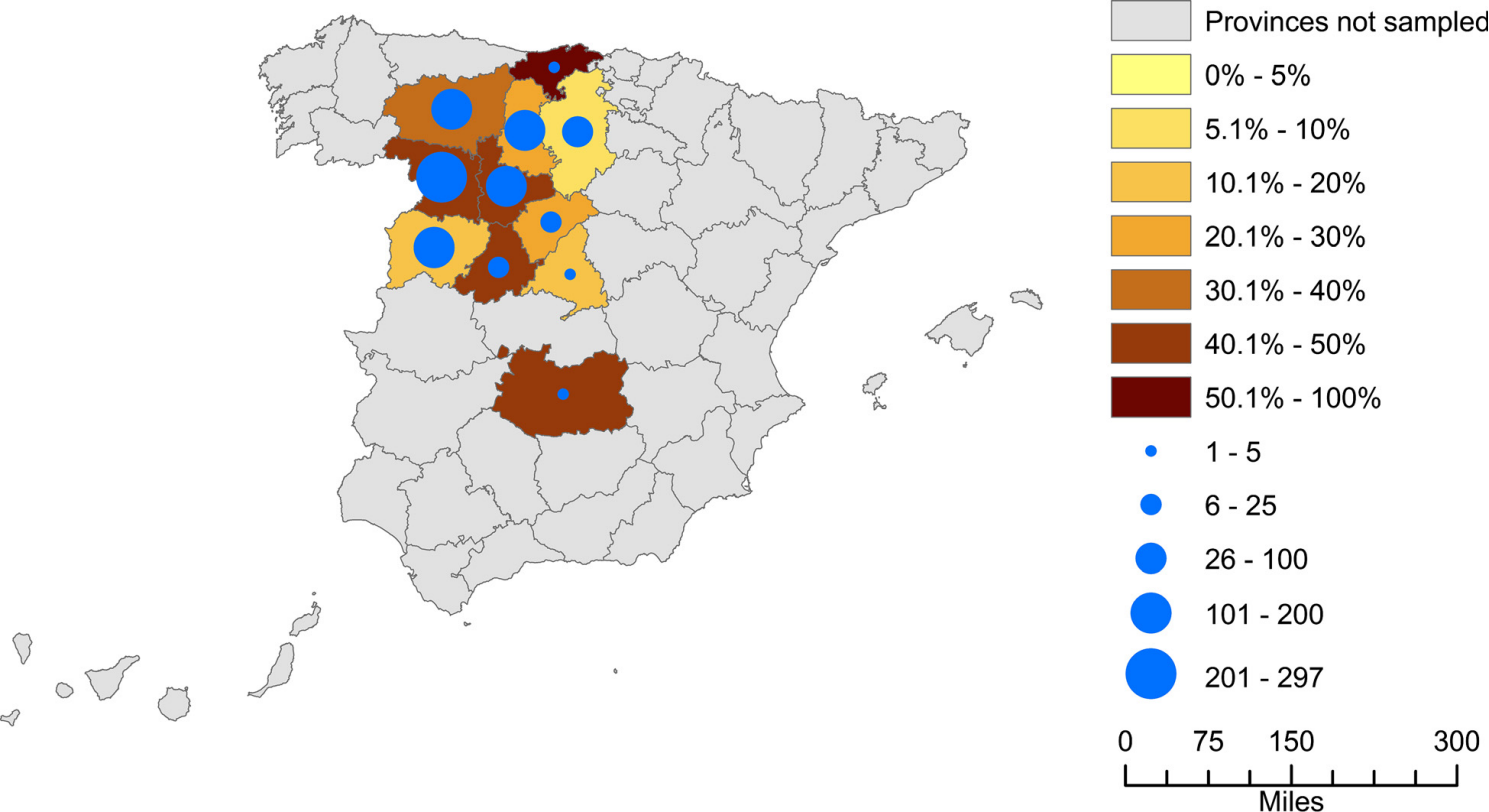
**Presence of *Mycoplasma agalactiae *per province over the 2008–2010 period.** The color code of each province indicates the presence rate of *M. agalactiae*. The circles indicate the number of dairy sheep farms sampled once in each province over the sampled period.

**Figure 2:**
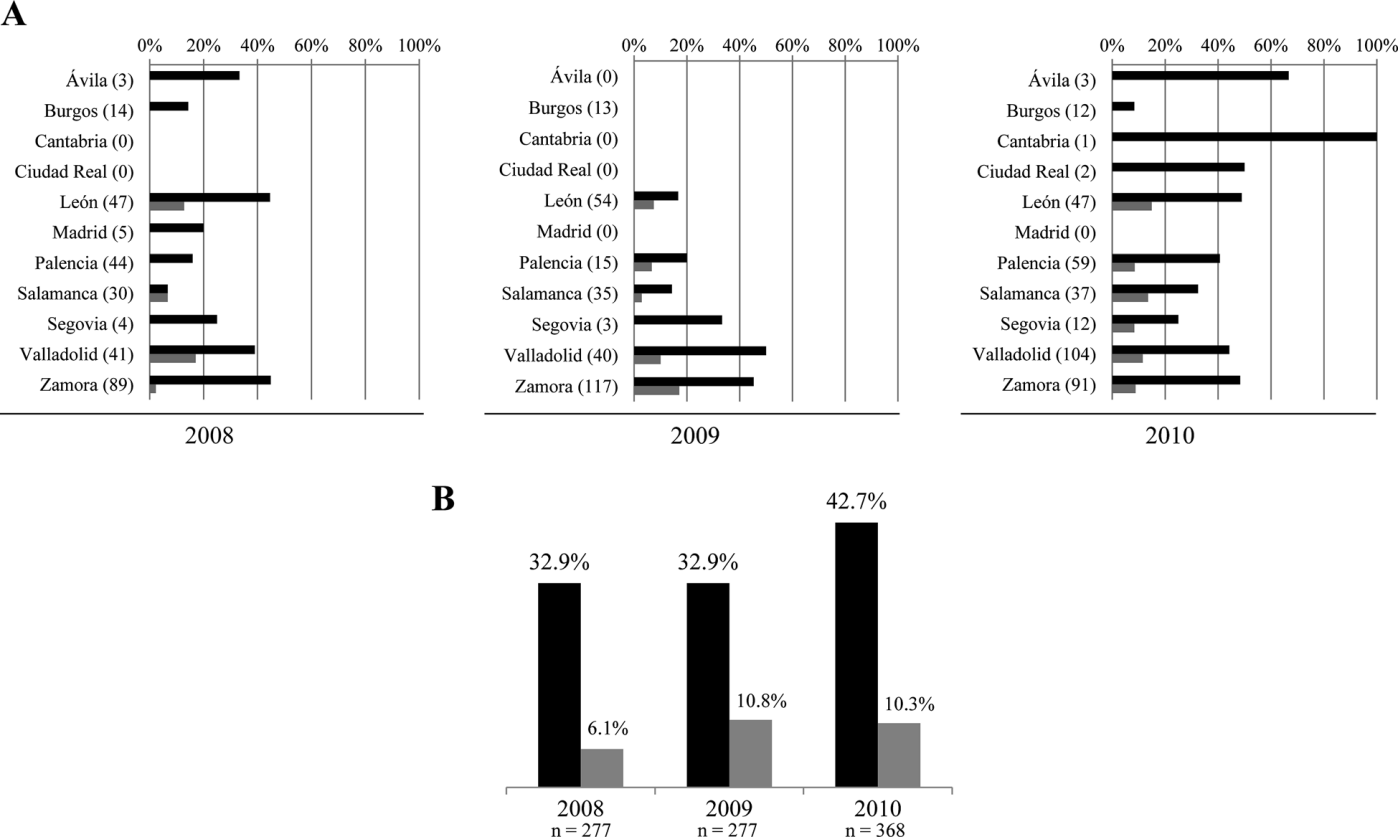
**Presence of *Mycoplasma agalactiae *in Spanish sheep farms in 2008, 2009 and 2010.** Black bars represent results by real time PCR and grey bars by microbiological identification. The numbers in brackets represent the number of sampled farms. **A**) Presence per province; **B**) presence per year.

Positive farms for *M*. *agalactiae* were located in all eleven provinces sampled (Figures 1, 2A). The frequency ranged from 7.7% to 100% of the flocks sampled per province revealing that the microorganism is widely distributed.

### Temporal distribution of *Mycoplasma agalactiae*

The presence of *M. agalactiae* increased between 2008 (32.9%) and 2010 (42.7%) (Figure 2B). The minimum and maximum frequency of detection of *M. agalactiae* ranged from 7.7% in February 2009 to 53.1% in May 2009. The months when the pathogen was most frequently detected were May 2009 (53.1%), July 2010 (51%) and October 2008 (50%). On the contrary, the lowest detection occurred in February 2009 (7.7%), June 2009 (14%) and April 2010 (16.7%). Thereby, 72 out of 194 farms (37.1%) were positive to *M*. *agalactiae* in Spring (i.e. farms analyzed in March, April and May), 113 out of 286 (39.5%) in Summer (i.e. June, July and August), 110 out of 280 (39.3%) in Autumn (i.e. September, October and November) and 44 out of 162 (27.2%) in Winter (i.e. December, January and February).

## Discussion

*M*. *agalactiae* was the only CA-causing *Mycoplasma* detected in dairy sheep farms located in the study area, and it was found to be widely distributed as it was detected in all eleven provinces sampled (Figure 1). In contrast, although *M*. *agalactiae* has been detected with a high prevalence in Spanish goat herds ranging from 40% to 66% [14,15], it seems that *M. mycoides* subsp. *capri* plays the major role in goat [2,16]. Interestingly, *M. capricolum* subsp. *capricolum* and *M. putrefaciens* have been recently isolated in goat herds located in the Canary Islands and Extremadura (southwestern Spain) regions [17,18], and yet those pathogens have not been detected in sheep flocks from the study area suggesting that Spanish dairy sheep are free of their presence or, at least, that their distribution would be restricted outside the sampling area. Furthermore, in this study we have observed that the frequency of detection of *M. agalactiae* increased from 32.9% in 2008 to 42.7% in 2010, suggesting that the pathogen has spread in the sampling area during the study period. On the other hand, we tried to establish relationships between the excretion of the microorganism in milk and the stages in sheep milk production (e.g. period of parturitions, the start of lactation) by analyzing the presence of *M. agalactiae* per month and per season in the different sampling years, but no pattern was found. We hypothesize that this is because the farms from the sampled region frequently had different production systems, and the same production stage may have occurred in a different time between farms.

To our knowledge, few surveys have been carried out internationally to determine which *Mycoplasma* species are circulating in sheep. In Northern Jordan, investigations revealed a seroprevalence in sheep, goat and mixed flocks of 25%, 21% and 30% respectively, for *M. agalactiae,* and 32%, 38% and 34% for *M. mycoides* subsp. *capri*, suggesting that both microorganisms are widely distributed in that country and to the same extent in sheep and goats [7,10]. This scenario is probably caused because in Jordan more than 93% of sheep and goats are kept together in mixed farms, so both hosts are exposed to the same pathogens. In contrast, the existence of mixed farms in Spain is not common, hindering the dissemination of other *Mycoplasma* species from goat herds to sheep flocks.

Detection of *M. agalactiae* by using microbiological techniques (9.2%) greatly underestimated its presence in comparison to detection by using real time PCR (36.8%). This finding prompts the recommendation that PCR-based methods should be routinely used in *Mycoplasma* detection, as microbiological identification leads to underestimation due to the fastidious growth requirements of mycoplasmas, and serology is not a suitable method in those areas, such as Spain, where systematic vaccination is extended.

The results of this study require careful interpretation. Firstly, this syndrome follows a chronic course in endemic areas and the animals present an intermittent excretion of the microorganism in milk [3,5]. Therefore, the presence of *M*. *agalactiae* could have been underestimated, as samples were collected from the milk tanks. In fact, the findings obtained in this study suggest a high level of intermittent excretion, as in more than 26% of the re-sampled farms a previously positive flock tested negative in a later sampling and 63.9% overall tested positive. Considering that *M. agalactiae* is a highly persistent pathogen, remaining in the animals for years [4,5], and that it is very difficult to eliminate from infected herds, we can assume that in most of those cases infected animals were not excreting the pathogen at the sampling time or that the number of bacteria present was below the limit of detection. Consequently, if monitoring of a herd needs be implemented, the inclusion of other suitable type of samples (e.g. blood, articular, auricular, ear canal, eye and vaginal swabs, nasal secretions, joint fluids) is strongly recommended. Secondly, the lower sensitivity of the conventional-PCR technique in comparison to the real time PCR could have underestimated the presence of the *Mycoplasma* species detected by the first method. Notwithstanding, all CA-causing mycoplasmas were detected after the inoculation of from 10^8^ to 10^2^ cells of each *Mycoplasma* spp. in 50 mL milk samples. Detection failed in samples containing 10^1^ cells (0.2 cells/mL) probably because the initial amount of mycoplasmas was not enough to compete during the enrichment step with all the other microbiota present in the milk samples. This suggests that all the PCR systems can detect the minimum quantity of mycoplasmas needed for growing in the enrichment step, and therefore that the presence of the *Mycoplasma* species as detected by conventional PCR would not be underestimated in detriment of the species detected by real time PCR. In addition, these results confirm that the enrichment step does not favor any species to the detriment to the others, and therefore that we can detect co-infected samples.

## Conclusions

This study provides for the first time an overview of the presence of CA-causing mycoplasmas circulating in Spanish dairy sheep farms. *M*. *agalactiae* was the only species detected in the study area showing a high frequency of presence, and it was found to be widely distributed. The high frequency of detection of the pathogen supports the implementation of control and prevention measures to hinder the dissemination of *M. agalactiae* and to prevent the entrance of other *Mycoplasma* species, such as systematic laboratory detection, isolation or slaughtering of infected flocks and tracing of livestock exchange.

## Methods

### Study area and sampling

From August 2008 to July 2009, and from April to December 2010, 1,798 samples were taken from 1,313 dairy sheep farms located in twelve provinces: Ávila, Burgos, Cáceres, Cantabria, Ciudad Real, León, Madrid, Palencia, Salamanca, Segovia, Valladolid and Zamora. Most provinces belong to the Spanish region which presents the highest sheep milk production (60% of total sheep milk) [19]. Selection of the farms was not performed following a formal randomization process, but blind samples were randomly provided by the Regional Interprofessional Dairy Laboratory. As blind sampling was carried out, 922 out of 1,313 farms were sampled once, and the resting 391 farms were sampled from 2 to 5 times. To ensure that no farms contribute more than once in the determination of the frequency of presence of the mycoplasmas, only those results obtained from the 922 farms sampled once have been used to perform the analyses showed in this study. The results obtained from the farms sampled several times have been only used to assess the level of intermittent excretion of the microorganisms in milk.

**Figure 3:**
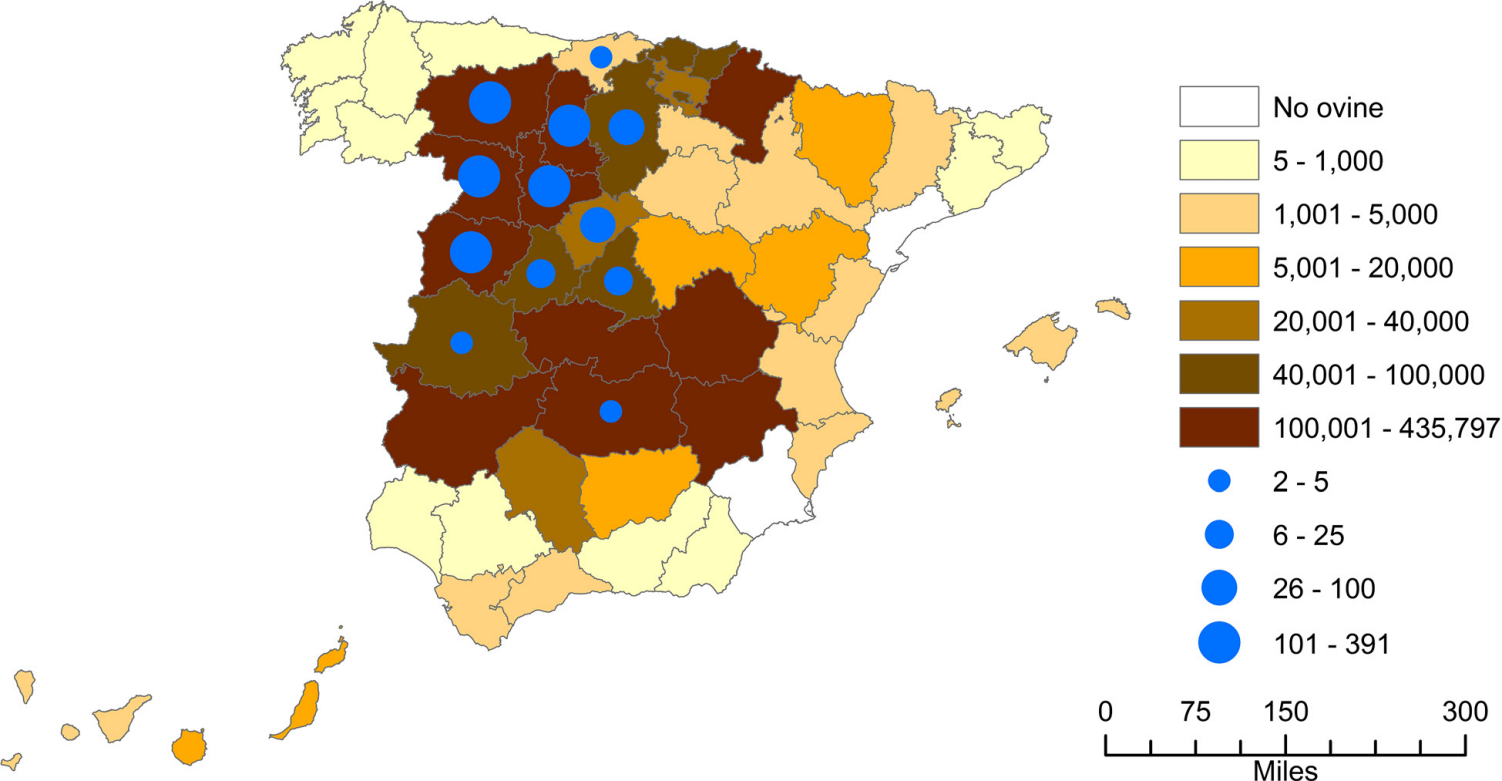
**Distribution of dairy sheep in Spain and number of farms sampled per province.** The color code of each province indicates the number of dairy sheep present in that province in 2012 ( http://www.marm.es/). The circles indicate the number of dairy sheep farms sampled in each province over the 2008–2010 period. Overall, 1,313 farms were sampled.

The sampling represents more than 16% of total dairy sheep farms in Spain and more than 44% of the farms located in the most productive region (Table 1). Location of the farms and overall number of dairy sheep per province is depicted in Figure 3. Each week, twenty raw milk samples of 50 ml were taken from the refrigerated tanks and preserved during transportation at 4°C with 133 μL of a bacteriostatic agent (9.975 × 10^-2^ μg chloramphenicol, 2.394 μg sodium azide, 1.33 μl ethanol, 5.985 μg trisodium citrate hydrate, 4.655× 10^-2^ μg bromophenol blue). The samples were immediately analyzed because freezing considerably reduces the viability of mycoplasmas [20].

**Table 1:** Geographic distribution of the Spanish dairy sheep farms sampled to detect CA-causing mycoplasmas

**Province**	**No. Farms**	**No. Sampled Farms**	**% Sampled Farms**
Ávila	91	9	9.9
Burgos	125	65	52.0
Cáceres	130	2	1.5
Cantabria	29	2	6.9
Ciudad Real	829	2	0.2
León	430	205	47.7
Madrid	174	6	3.4
Palencia	414	177	42.8
Salamanca	384	132	34.4
Segovia	79	30	38.0
Valladolid	491	292	59.5
Zamora	902	391	43.3
**12 provinces**	**4,078**	**1,313**	**32.2**

Study areas for detecting the presence of CA-causing mycoplasmas in Spanish dairy sheep farms. The samples were collected from flocks located in twelve provinces showing highest sheep milk production in Spain.

### Culture of milk samples and DNA extraction

One hundred microlitres of the milk samples were cultivated in 9.9 ml of Mycoplasma broth base with Mycoplasma supplement G (Oxoid, Hampshire, UK), at 37°C in 5% CO_2_ atmosphere for 3 days. For DNA extraction, 1 ml of culture was centrifuged at 13,000 × *g* for 15 min, and the cell pellet was resuspended in 100 μl of 10 mM Tris–HCl (pH 8.0) for further incubation at 95°C with shaking for 20 min. Then, samples were centrifuged at maximum speed for 3 min, the supernatant was transferred into new tubes, and subsequently analyzed for CA-causing mycoplasmas.

### Molecular detection of CA-causing mycoplasmas by PCR-based methods

Since *M. agalactiae* is the main aetiological agent, its presence was assessed in all farms. The presence of *M. mycoides* subsp. *capri*, *M. capricolum* subsp. *capricolum* and *M. putrefaciens* was evaluated in 597 samples taken from 528 sheep farms distributed in the eleven provinces. The latter samples were collected in July 2009 and since April to December 2010. Molecular detection of *M*. *agalactiae* was conducted by real time PCR as previously described [11] in a 7500 real time PCR system platform (Applied Bios stems, Carlsbad, CA, USA). *M. mycoides* cluster and *M. putrefaciens* detection was carried out by conventional PCR [12,13]. PCR amplification products were resolved by gel electrophoresis on 1.5% (w/v) agarose gels and visualized after staining with ethidium bromide by using a UV transilluminator (BioRad, Hercules, CA, USA). All samples were analyzed in duplicate and positive, negative extraction and non template controls were included in each run. The limit of detection of the real time PCR system corresponds to approximately 1 genomic equivalent per reaction. Those of the conventional PCR systems have not been reported. Therefore, in order to demonstrate that the PCR methods used in this study could detect the same levels of *Mycoplasma* contamination, and that detection of naturally co-infected samples would be possible, raw milk samples were artificially contaminated with decreasing concentrations of all four mycoplasmas and then processed following the protocol detailed above.

### Microbiological detection

Pre-enriched milk samples were cultured in agar plates of the selective medium, which were incubated at 37°C in a 5% CO_2_ atmosphere for 3 to 4 days. After incubation, colonies consistent with *Mycoplasma* phenotype were identified using an optical microscope Leica DMLS under 100 × magnifications (LeicaMicrosystems GmbH, Wetzlar, Germany).

### Isolation of mycoplasmas

We isolated from 1 to 6 colonies from each positive milk sample and subculture them in specific broth medium at 37°C, in a 5% CO_2_ atmosphere for 3 days. The nature of the isolates was reconfirmed by real time PCR [11] and the purity of the culture by checking the morphology of the colonies on agar plates of the selective medium. Finally, the isolates were cryopreserved in glycerol (16% v/v) and stored at −80°C.

## Abbreviations

CA, Contagious Agalactia.

## Competing interests

The authors declare that they have no competing interests.

## Authors’ contributions

JA carried out the experiments, results analysis and drafted the manuscript. DRL and MH led the project, designed the study and revised the results and manuscript. All authors read and approved the final manuscript.
